# “Qi Nan” agarwood restores podocyte autophagy in diabetic kidney disease by targeting EGFR signaling pathway

**DOI:** 10.1186/s13020-024-00923-z

**Published:** 2024-04-23

**Authors:** Ning Li, Xuenan Liu, Hao Wang, Yingling Duan, Yu Zhang, Ping Zhou, Haofu Dai, Tian Lan

**Affiliations:** 1 Department of Pharmacology, School of Pharmacy, Guangdong Pharmaceutical University, No. 280 Wai Huan Dong Road, Guangzhou, 510006 China; 2 National Key Laboratory for Tropical Crop Breeding, Institute of Tropical Bioscience and Biotechnology, International Joint Research Center of Agarwood, Hainan Engineering Research Center of Agarwood, Chinese Academy of Tropical Agricultural Sciences, No. 4 Xue Yuan Road, Haikou, 571101 China; 3 Department of Pharmacology, College of Pharmacy, Harbin Medical University, Harbin, 150086 China; 4 Department of Pediatric Nephrology and Rheumatology, Sichuan Provincial Maternity and Child Health Care Hospital, Sichuan Clinical Research Center for Pediatric Nephrology, 290 Shayan West Second Street, Wuhou District, Chengdu, 610045 Sichuan China

**Keywords:** DKD, “QN” agarwood, Podocytes, Autophagy, EGFR

## Abstract

**Background:**

Diabetic kidney disease (DKD) is a microvascular complication of diabetes mellitus, contributing to end-stage renal disease with limited treatment options. The development of DKD is attributed to podocyte injury resulting from abnormal podocyte autophagy. Consequently, the restoration of podocyte autophagy is deemed a practicable approach in the treatment of DKD.

**Methods:**

Diabetic mice were induced by streptozotocin and high-fat diet feeding. Following 8 weeks of “QN” agarwood treatment, metrics such as albuminuria, serum creatinine (Scr), and blood urea nitrogen (BUN) were evaluated. Renal histological lesions were evaluated by H&E, PAS, Masson, and Sirius red staining. Evaluation of the effects of “QN” agarwood on renal inflammation and fibrosis in DKD mice through WB, q-PCR, and IHC staining analysis. Cytoscape 3.7.1 was used to construct a PPI network. With the DAVID server, the gene ontology (GO) functional annotation and the Kyoto encyclopedia of genes and genomes (KEGG) signaling pathways of the target enrichment were performed. Molecular docking and binding affinity calculations were conducted using AutoDock, while PyMOL software was employed for visualizing the docking results of active compounds and protein targets.

**Results:**

The results of this study show that “QN” agarwood reduced albuminuria, Scr, and BUN in DKD mice, and improved the renal pathological process. Additionally, “QN” agarwood was observed to downregulate the mRNA and protein expression levels of pro-inflammatory and pro-fibrotic factors in the kidneys of DKD mice. Network pharmacology predicts that “QN” agarwood modulates the epidermal growth factor receptor (EGFR) signaling pathway. “QN” agarwood can increase the expression of LC3B and Nphs1 in DKD mice while reducing the expression of EGFR.

**Conclusion:**

The present study demonstrated that “QN” agarwood ameliorated renal injury in DKD by targeting EGFR and restoring podocyte autophagy.

**Graphical Abstract:**

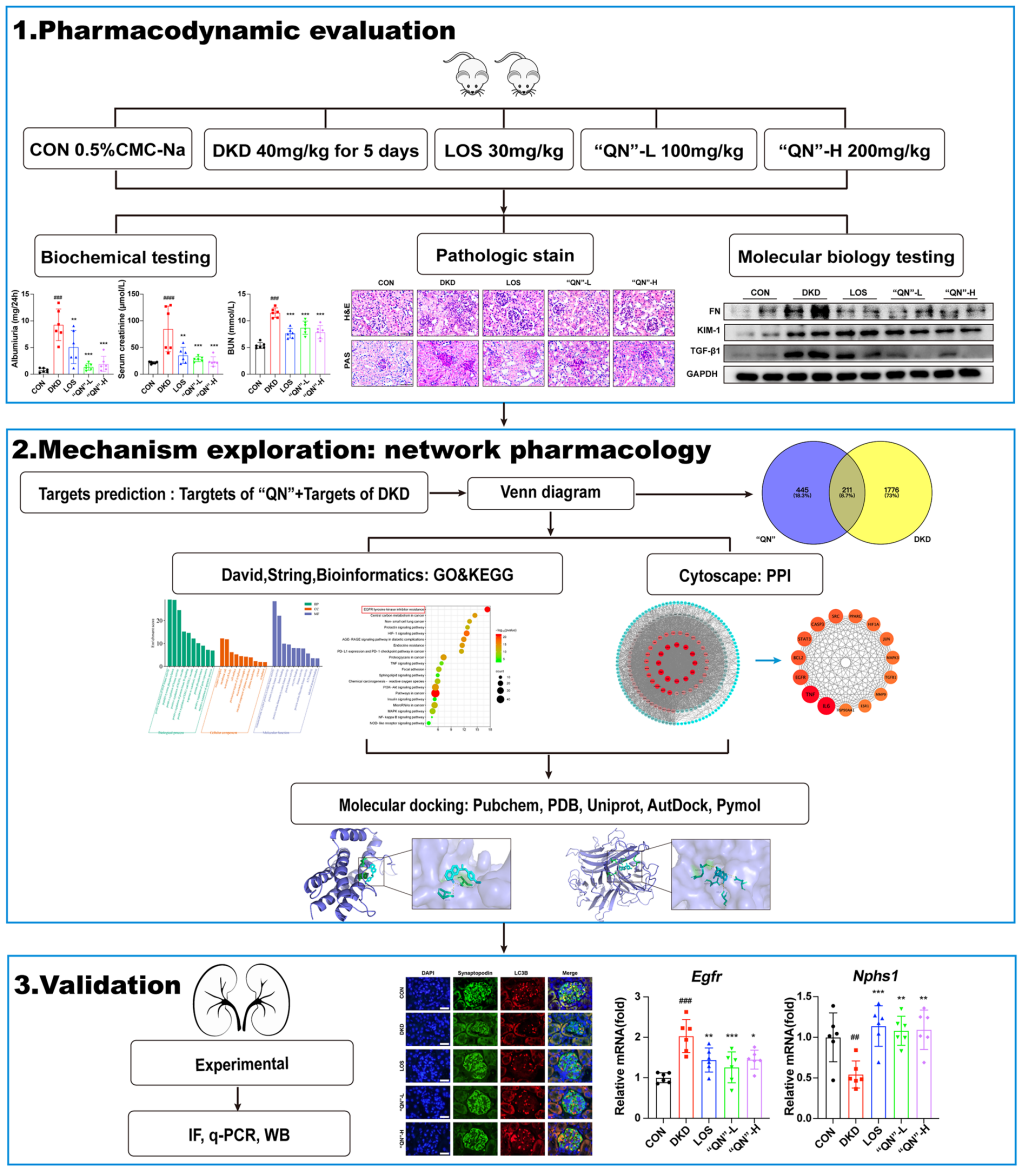

## Introduction

Diabetes mellitus is a persistent chronic endocrine disorder, also termed as “Xiao Ke” in Chinese medicine [1]. According to data from the International Diabetes Federation (IDF), estimated at approximately 40% of individuals diagnosed with diabetes mellitus are prone to developing kidney dysfunction that may eventually progress to renal failure [2]. DKD is characterized by its persistent albuminuria, impaired glomerular filtration rate (GFR), and progressively deteriorating renal function [3]. Podocytes serve as the primary constituent of the glomerular filtration barrier structure [4, 5]. Podocyte damage, apoptosis, and shedding are important causes of continued albuminuria, contributing to the relentless aggravation of DKD [6]. It should be emphasized that podocytes are highly specific, terminally differentiated visceral epithelial cells that have little ability to proliferate and repair once damaged and lost. Autophagy, a highly conserved cellular process, sequesters damaged proteins and organelles, delivering them to lysosomes for decomposition, and plays a crucial role in mediating intracellular recycling, organelle renewal, and cellular homeostasis maintenance [7]. Mounting evidence suggests that podocytes exhibit a heightened intensity of basal autophagy under normal physiological circumstances compared to other inherent renal cells [8], and this elevated intensity of autophagy in podocytes serves as a crucial protective against high glucose conditions and is essential for maintaining their normal physiological function [9].

Currently, clinical treatments for DKD primarily include hypoglycemic, lipid-lowering, antihypertensive, and anti-inflammatory therapies. Regrettably, there is a dearth of dedicated pharmaceutical agents for the treatment of DKD, and symptomatic treatment is the mainstay, and the synthetic drugs employed to treat DKD presenting numerous adverse effects [10]. Natural products derived from Chinese herbal medicine have long been regarded as potential sources of drugs to treat a variety of serious diseases, and effects by interacting with multiple targets, offering relative safety and lower toxicity, thereby impeding disease progression [11]. Therefore, some phytochemicals may be effective kidney protectors. Agarwood, derived from the *Thymelaeaceae* family’s *Aquilaria* and *Gyrinops* species, is produced as a consequence of physical stem damage or pathogenic infestation [12]. Agarwood, a traditional drug, has been historically employed for the treatment of diverse ailments such as rheumatism, arthritis, body aches, asthma, hyperuricemia, and wind repellent [13]. “QN” agarwood, an exceptionally scarce and valuable type of agarwood, commands prices hundreds or even thousands of times higher than ordinary agarwood [14]. “QN” agarwood significantly activated the AMPK pathway in HEK292T cells and strengthened the phosphorylation of AMPK, and the Flindersia-type 2-(2-phenylethyl)chromones in the ethanolic extract of “QN” agarwood may be an excellent precursor for the development of therapeutic drugs for metabolic syndrome [15].

Network pharmacology integrates molecular biology, pharmacology, electronic technology, and bioinformatics to investigate the intricate interactions between drugs and biological systems, elucidating how drugs effectively treat diseases at the biological target and pathway levels [16]. The diverse components, multiple targets, and broad-spectrum efficacy of traditional Chinese medicine align closely with the fundamental principles of network pharmacology [17, 18]. Currently, network pharmacology has gained extensive utilization in deciphering the underlying mechanisms of physiological activity of Chinese medicines [19]. However, there are no comprehensive network pharmacologic studies investigating the effects of “QN” agarwood against DKD.

The objective of this study is to explore the effects of “QN” agarwood on DKD and utilize network pharmacology techniques to further elucidate its underlying mechanism of action in DKD. The outcomes of this research aim to provide valuable insights for future pharmacological investigations and clinical management of DKD.

## Materials and methods

### Materials and reagents

“QN” agarwood was harvested from the 12 months artificial holing of *Aquilaria sinensis* “Reke No. 2” in Dianbai, Guangdong province in May 2020. The original plant material of “QN” agarwood was identified by Prof. Haofu Dai, affiliated with the Institute of Tropical Bioscience and Biotechnology, Chinese Academy of Tropical Agricultural Sciences. It is extracted by refluxing 95% ethanol. Streptozotocin (STZ) and Sirius red staining solution were obtained from Sigma-Aldrich (St Louis, USA). The high-fat diet was acquired from the Guangdong Experimental Animal Centre (Guangzhou, China). Losartan (LOS) was acquired from MSD (J20200067, Kenneworth, USA). CMC-Na was acquired from Sangon Biotech (9025-26-8, Shanghai, China). Serum creatinine, albuminuria, and blood urea nitrogen determination kits were purchased from Nanjing Jiancheng (Nanjing, China). Hematoxylin and eosin (H&E) and Masson staining kits were acquired from Leagene (Beijing, China). The BCA kit was acquired from MEIMIAN (Jiangsu, China). SYBR Green supermix was acquired from BioRad (CA, USA). Trizol was acquired from Trans-Gen Biotech (Beijing, China). Anti-fibronectin, Anti-KIM-1, and Anti-TGF-β1 antibodies were procured from BOSTER (Wuhan, China). Anti-Ccl2 antibodies were acquired from Santa (Dallas, USA). Anti-EGFR and anti-Synaptopodin antibodies were purchased from Proteintech (Chicago, USA). Anti-GAPDH antibodies were acquired from Trans-Gen Biotech (Beijing, China). Anti-LC3B antibodies and Anti-IL6 antibodies were obtained from Abmart (Shanghai, China). Horseradish peroxidase (HRP)-conjugated Affinity Pure goat anti-mouse IgG and anti-rabbit IgG were procured from Zhongshan Golden Bridge Biotechnology (Beijing, China).

### Laboratory animal preparation

Specific pathogen-free (SPF) C57BL/6 male mice (5 weeks old, weighing 18–20 g) were procured from Beijing Viton Lever Biotechnology (SCXK 2021-0057, Beijing, China). The mice were accommodated in the Animal Experimentation Center of Guangdong Pharmaceutical University. The facility ensured a controlled environment with an ambient temperature ranging from 20–25 °C, humidity between 55 and 65%, and a 12-h light–dark cycle for the mice. During the study, the mice were granted unrestricted access to both water and food.

### Establishment of DKD mouse model and grouping of drug administration

Six mice were randomly assigned according to body weight and given ordinary food, and the rest mice were pre-fed with HFD for one week. After fasting for 12 h, the mice were administered intraperitoneal injections of STZ at a dosage of 40 mg/kg once daily for five consecutive days [20], the control group received intraperitoneal injections of an equivalent dose of sodium citrate buffer. One week later, fasting blood glucose (FBG) levels were evaluated to verify the established diabetes model. Mice with FBG standards reached or surpassed 11.1 mM were considered as diabetic. The mice were randomly assigned to the following groups (Fig. 2A): Control group (CON, 0.5% CMC-Na); DKD model group (DKD, 0.5% CMC-Na); Positive control/Losartan group (LOS, 30 mg/kg/d); Low-dose “QN” agarwood group (“QN”-L, 100 mg/kg/d); High-dose “QN” agarwood group (“QN”-H, 200 mg/kg/d). All groups received daily oral administration of 0.5% CMC-Na, “QN” agarwood, or Losartan for 8 weeks.

### Drug targets of “QN” agarwood

The key active components in “QN” agarwood were reported in previous study [14]. The Canonical SMILES numbers of the components were acquired using the PubChem website (https://pubchem.ncbi.nlm.nih.gov) (queried on 20 September 2023). Additionally, the Swiss Target Prediction database (http://www.swisstargetprediction.ch) (queried on 20 September 2023) was utilized to predict the targets of each ingredient. Subsequently, the identified targets were uploaded to the Uniprot database (https://www.uniprot.org) for gene name normalization and removal of duplicates, resulting in the acquisition of the final targets.

### Therapeutic targets for DKD

To retrieve the disease targets related to DKD, a keyword search for “Diabetic kidney disease” was conducted in three databases: Online Mendelian Inheritance in Man database (https://www.omim.org), Gene Cards database (https://www.genecards.org), and Therapeutic Target Database (http://db.idrblab.net/ttd). To acquire the therapeutic targets of action, the identified targets were combined, and any duplicate entries were removed (queried on 25 September 2023).

### Protein–protein interaction (PPI) network

The Venny2.1.0 website (queried on 25 September 2023) was used to construct a Venn diagram of “QN” agarwood active components and disease targets to obtain intersecting targets for further analysis. The obtained intersection targets were uploaded to the STRING database (http://cn.string-db.org) (queried on 25 September 2023). Cytoscape 3.7.1 software was utilized to analyze the node degree values, and the twofold median was filtered to show 15 targets.

### Kyoto encyclopedia of genes and genomes (KEGG) and gene ontology (GO) enrichment analysis

To further elucidate the mechanism of “QN” agarwood for the treatment of DKD, the obtained core targets were uploaded to the David database (https://david.ncifcrf.gov/summary.jsp) (queried on 25 September 2023). Identifier was selected as OFFICIAL-GENE-SYMBOL and the species as HOMO. KEGG pathway analysis was utilized for enrichment analysis. GO functional enrichment analysis was conducted using the GOTERM-BP-DIRECT, GOTERM-CC-DIRECT, and GOTERM-MF-DIRECT methods to identify enriched biological processes, cellular components, and molecular functions, respectively. The enrichment results for the core targets were visualized using the bioinformatics platform.

### Molecular docking between “QN” agarwood and targets

Retrieve the 3D structures of the active components from PubChem (enquired on 25 September 2023). Retrieve the 3D structure of the target protein from the PDB database (queried on 25 September 2023). Autodock software processing was utilized to predict the binding ability of the active ingredient to the target protein. The docking model with the lowest binding energy was recorded and the corresponding data were saved for visual presentation in Pymol.

### Quantitative real-time PCR (q-PCR)

A total of 20 mg of kidney tissue was collected and homogenized with Trizol. After the addition of chloroform to the homogenate, it was subjected to centrifugation. The RNA-containing supernatant was extracted meticulously and mixed with an equal volume of isopropanol. Subsequently, the mixture underwent high-speed centrifugation at 4 °C to obtain a precipitate, which was then rinsed with 75% ethanol. The resulting RNA precipitate was dissolved in DEPC water. The concentration of total RNA was assessed by utilizing a UV spectrophotometer to measure the absorbance ratios at 260 and 280 nm. Reverse mRNA transcription into cDNA was provided with the reverse transcription kit. The resulting cDNA was subjected to q-PCR analysis using SYBR Green Supermix and specific primers. The relative expression levels of each mRNA were calculated using the comparative threshold cycle (Ct) method, with the Ct values normalized to the reference gene GAPDH. The list of primers can be found in Table 1.

**Table 1:** Primer Sequences for q-PCR

Genes	Forward primer	Reverse primer
*Il-6*	TAGTCCTTCCTACCCCAATTTCC	CTGTTGTTCAGACTCTCTCCCT
*Gapdh*	AGGAGTAAGAAACCCTGGAC	CTGGGATGGAATTGTGAG
*Kim-1*	ACATATCGTGGAATCACAACGAC	ACTGCTCTTCTGATAGGTGACA
*Ccl2*	TTAAAAACCTGGATCGGAACC	GCATTAGCTTCAGATTTACG
*Fn1*	GTGGCTGCCTTCAACTTCTC	AGTCCTTTAGGGCGGTCAAT
*Tnfα*	CCCTCACACTCAGATCATCTTCT	GCTACGACGTGGGCTACAG
*Tgfb1*	GTGGAAATCAACGGGATCAG	ACTTCCAACCCAGGTCCTTC
*β-actin*	GTGACGTTGACATCCGTAAAGA	GCCGGACTCATCGTACTCC
*Egfr*	TGTGGGCCTGACTACTACGA	TGGGTCTAGAGGAGGAGTGC
*Col4a1*	ATCGGATACTCCTTCCTCATGC	CCAGGGGAGACTAGGGACTG
*Nphs1*	TCACCGTGAATGTTCTGTTCC	AGTGTGGCTAAGGGATTACCC

### Western blotting (WB) analysis

The total protein from kidney tissue was extracted with RIPA lysis buffer supplemented with 1% PMSF, followed by homogenization and centrifugation at 12,000 rpm, 4 °C for 30 min. The resulting supernatant was collected, and its protein content was quantified using a BCA kit. After buffer addition, the proteins were subjected to denaturation by boiling in a metal bath at 100 °C for 10 min. Following SDS-PAGE separation, each sample was subsequently transferred to a polyvinylidene fluoride membrane. After the membranes were closed with 10% non-fat dry milk powder (formulated using TBST included 0.1% Tween-20) for 1 h, they were subjected to overnight primary antibody exposure at 4 °C. Subsequently, the membranes underwent a 1 h interaction with horseradish peroxidase-conjugated secondary antibodies. Chemical imaging was performed with the ultra-sensitive ECL chemiluminescent reagent. The bands were quantified and counted using ImageJ software.

### Detection of biochemical parameters

Mice were anesthetized through intraperitoneal injection of a 5% solution of chloral hydrate. Blood samples were left at room temperature for approximately 30 min, and then subjected to centrifugation at 3000 rpm for 15 min to collect the serum. Renal function-related parameters, including serum creatinine (Scr), blood urea nitrogen (BUN), and albuminuria levels, were measured according to the manufactory’s instructions.

### Immunohistochemistry (IHC) and immunofluorescence (IF)

The kidney sections were prepared, rehydrated, and subjected to immunohistochemical analysis by incubating them with anti-CD68 antibodies. Additionally, kidney tissue sections were co-stained with LC3B and Synaptopodin, and fluorescence microscopy was utilized to capture images. The positively stained areas were then quantified and measured using ImageJ software.

### Renal histopathological analysis

Following 8 weeks of drug administration, the renal tissues were longitudinally sectioned, fixed in a 4% paraformaldehyde solution for 24 h, and thereafter encased in paraffin. H&E staining was employed to visualize and examine the tissue structure. Masson trichrome stain and Sirius red staining were utilized to assess the degree of renal fibrosis [21, 22]. The quantification of positive fibrotic areas was performed using ImageJ software.

### Quantitative and statistical analysis

The data were expressed as mean ± standard deviation (SD). Statistical significance was evaluated utilizing GraphPad Prism 9.0 software. Significance was established using two-tailed Student’s t test and one-way analysis of variance (ANOVA) with Tukey’s multiple comparisons test. *P*<0.05 was considered to be a significant difference.

## Results

### “QN” agarwood mitigates renal impairment in diabetic mice

**Fig. 1:**
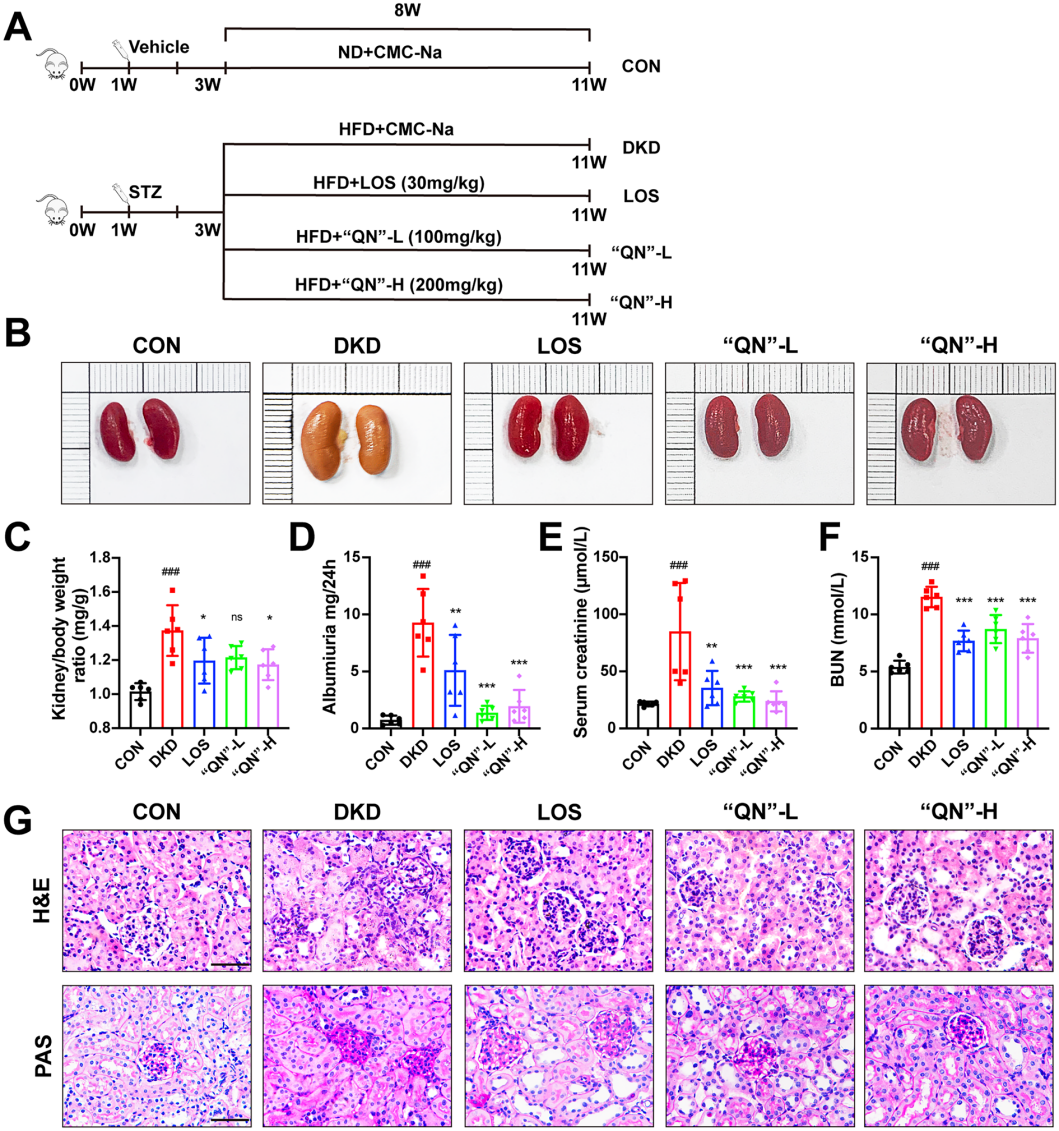
“QN” agarwood mitigates renal injury in diabetic mice. **A** Schematic study framework. **B** A representative sample of kidneys was selected from each group. **C** Kidney-to-Body Weight Ratios (mg/g). **D** Albuminuria (mg/24 h). **E** Serum creatinine (µmol/L) was measured. **F** BUN (mmol/L). **G** Representative images of renal tissue stained with H&E were captured, Scale bar = 40X.^*###*^*p* < 0.001 versus CON group; ^*^p < 0.05, ^**^p < 0.01, ^***^p < 0.001 versus DKD group. n.s indicates no statistically significant difference

To investigate the renoprotective effects of “QN” agarwood on diabetic mice, DKD mice were induced by HFD/STZ for 8 weeks. (Fig. 1A). “QN” agarwood successfully alleviated the elevation of 24 h albuminuria, Scr, BUN in DKD mice (Fig. 1D-F), and the kidney-to-body weight ratios (Fig. 1B,C). In addition, H&E and PAS stainings showed that the morphological changes and glycogen deposition of glomeruli were also improved by the treatment of “QN” agarwood (Fig. 1G). The above results indicate that “QN” agarwood has a potential protective effect on kidney damage in DKD mice.

### “QN” agarwood mitigated renal inflammation and fibrosis in mice with DKD

**Fig. 2:**
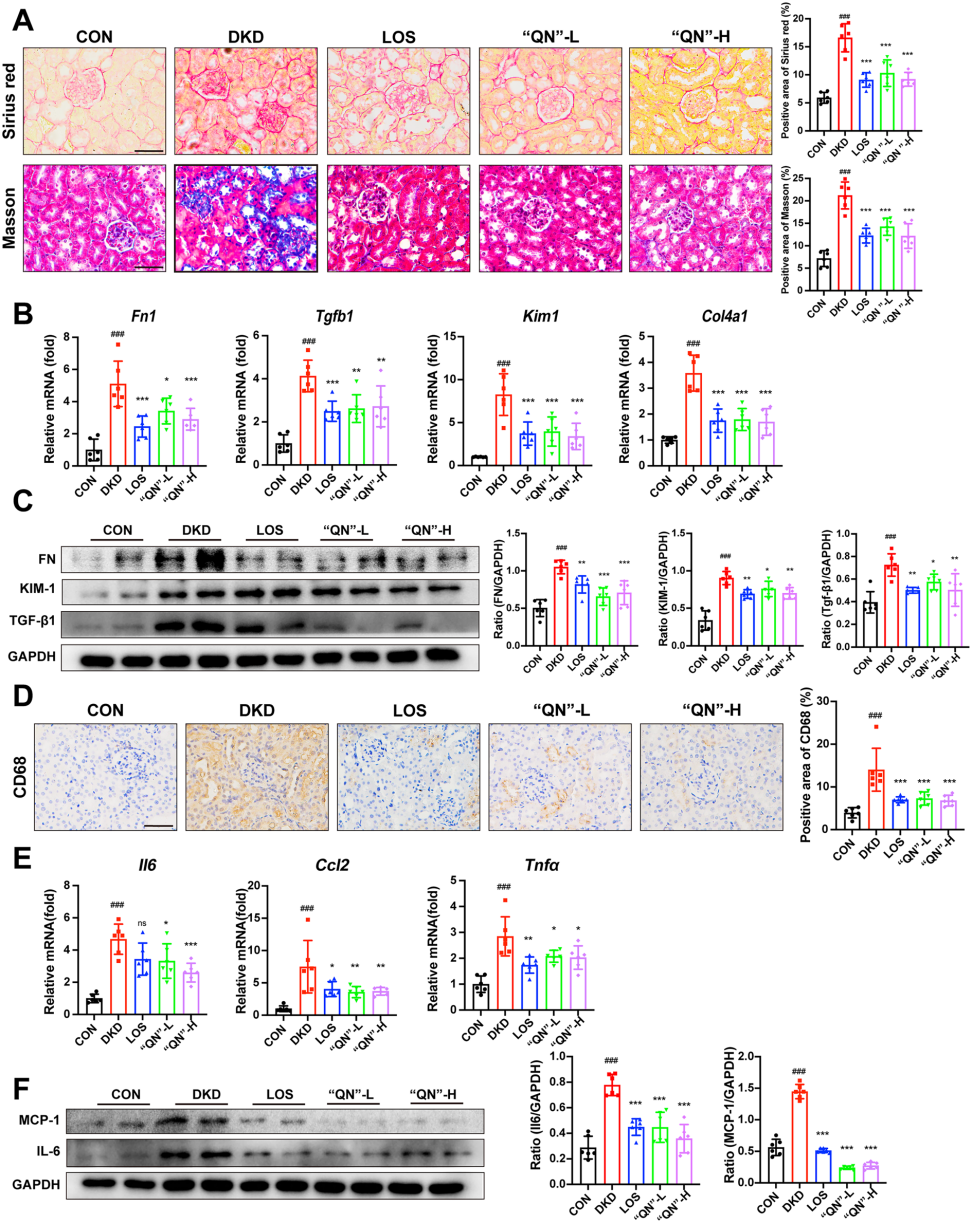
“QN” agarwood mitigated renal inflammation and fibrosis in mice with DKD. **A** Representative Images of Collagen Staining in Renal Tissue: Masson Staining and Sirius Red Staining, Scale bar = 40X. **B** The expression levels of *Fn1*, *Col4a1*, *Kim1*, and *Tgfb1* mRNA in the renal tissue were assessed. **C** Protein expression levels of FN, KIM-1, and TGF-β1 in the kidney were examined using WB analysis. **D** Representative images of renal tissue were obtained through immunohistochemical staining for CD68, Scale bar = 40X. **E** The expression levels of *Ccl2*, *Il6*, and *Tnfα* mRNA in the renal tissue were assessed. **F** WB of MCP-1 and IL6 in the kidney. ^*###*^*p* < 0.001 versus CON group; ^*^p < 0.05, ^**^p < 0.01, ^***^p < 0.001 versus DKD group. n.s indicates no statistically significant difference

Masson staining and Sirius Red staining revealed a pronounced reduction in the accumulation of collagen fibers within the renal interstitium following the administration of “QN” agarwood (Fig. 2A). The results obtained from q-PCR and WB analysis provided additional evidence supporting the ability of “QN” agarwood to mitigate renal fibrosis (Fig. 2B, C). The infiltration of CD68-positive in the kidney was quantified, and it was observed that treatment with “QN” agarwood significantly inhibited their infiltration (Fig. 2D). The findings demonstrated that “QN” agarwood treatment significantly decreased the pro-inflammatory cytokines C-C motif chemokine ligand 2 (Ccl2), interleukin 6 (IL6), and tumor necrosis factor-α (TNF-α) mRNA levels and protein expression (Fig. 2E, F). These results demonstrated that “QN” agarwood alleviate the occurrence of renal inflammation and fibrosis.

### Common targets of the “QN” agarwood active ingredient and DKD

**Fig. 3:**
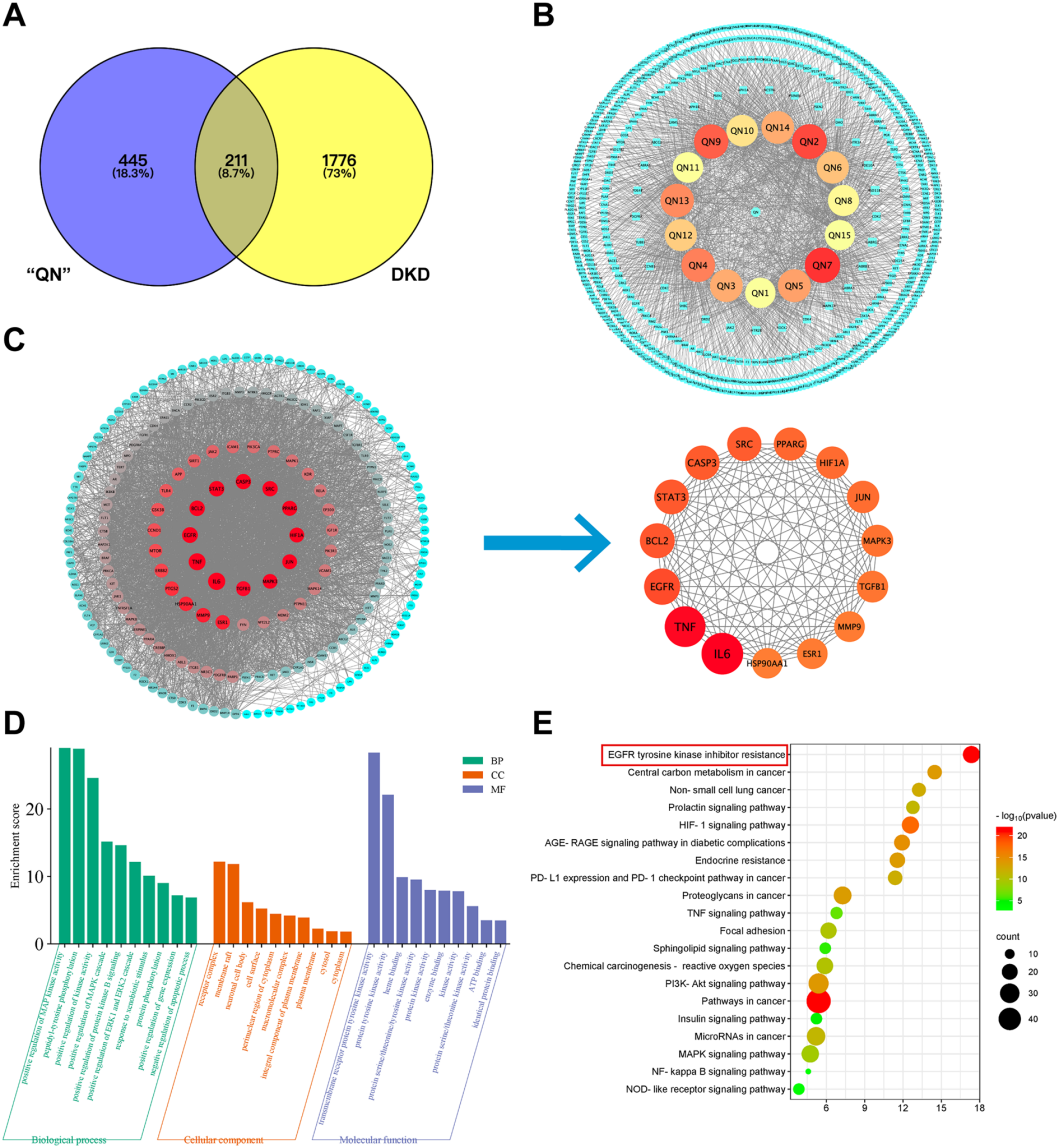
Identification of “QN” agarwood anti-DKD therapeutic targets. **A** Venn diagram of “QN” agarwood and DKD therapeutic targets. **B** “Active Ingredient-Target” network. **C** Screening of the key targets in the PPI network of “QN” agarwood and DKD. **D** GO enrichment analysis of “QN” agarwood components with DKD core targets. **E** KEGG enrichment analysis of “QN” agarwood components with DKD core targets

The Swiss Target Prediction database was utilized to search for the drug target was performed on the 15 qualified bioactive ingredients selected from “QN” agarwood in previous literature reports (Table 2) and 656 targets were obtained after summarizing the 15 “QN” agarwood active ingredient targets and removing duplicates. The potential targets of DKD were gathered from the Gene Cards database, OMIM database, and TTD database, and 1987 targets were obtained after de-duplication. The intersection of “QN” agarwood active ingredient targets and DKD targets yielded 211 collaborative targets, which were latent anti-DKD targets of the “QN” agarwood active ingredient (Fig. 3A).

**Table 2:** “QN” agarwood active ingredients

Abbreviation	Full name		Canonical SMILES
“QN”1	2-[(4-methoxybenzoyloxy)methyl]chromone		COC1=CC=C(C=C1)C(=O)OCC1=CC(=O)C2=C(O1)C=CC=C2
“QN”2	2-(2-phenylethyl)chromone		C1=CC=C(C=C1)CCC2=CC(=O)C3=CC=CC=C3O2
“QN”3	2-[2-(3-hydroxy-4-methoxyphenyl)ethyl]chromone		COC1=C(O)C=C(CCC2=CC(=O)C3=C(O2)C=CC=C3)C=C1
“QN”4	2-[2-(3-methoxy-4-hydroxyphenyl)ethyl]chromone		COC1=C(O)C=CC(CCC2=CC(=O)C3=C(O2)C=CC=C3)=C1
“QN”5	2-[2-(4-methoxyphenyl)ethyl]chromone		COC1=CC=C(CCC2=CC(=O)C3=C(O2)C=CC=C3)C=C1
“QN”6	2-[2-(2-hydroxyphenyl)ethyl]chromone		OC1=C(CCC2=CC(=O)C3=C(O2)C=CC=C3)C=CC=C1
“QN”7	2-[2-(4-hydroxyphenyl)ethyl]chromone		OC1=CC=C(CCC2=CC(=O)C3=C(O2)C=CC=C3)C=C1
“QN”8	6-hydroxy-2-[2-(3-hydroxy-4-methoxyphenyl)ethyl]chromone		COC1=C(O)C=C(CCC2=CC(=O)C3=C(O2)C=CC(O)=C3)C=C1
“QN”9	6-methoxy-2-[2-(4-methoxyphenyl)ethyl]chromone		COC1=CC=C(CCC2=CC(=O)C3=C(O2)C=CC(OC)=C3)C=C1
“QN”10	methyl 4-hydroxybenzoate		COC(=O)C1=CC=C(C=C1)O
“QN”11	(*E*)-anethole		CC=CC1=CC=C(C=C1)OC
“QN”12	7β-*H*-9(10)-ene-11,12-epoxy-8-oxoeremophilane		C[C@H]1CCCC2=CC(=O)[C@@H](C[C@]12C)C1(C)CO1
“QN”13	(5*S*,7*S*,9*S*,10*S*)-(−)-9-hydroxy-selina-3,11-dien-14-al		[H][C@@]12C[C@@H](C[C@H](O)[C@@]1(C)CCC=C2C=O)C(C) =C
“QN”14	β-elemen-9β-ol		CC(=C)[C@H]1C[C@@H](O)[C@@](C)(C=C)[C@H](C1)C(C)=C
“QN”15	squalene		CC(=CCCC(=CCCC(=CCCC=C(C)CCC=C(C)CCC=C(C)C)C)C)C

### Intersecting targets (PPI) Network and core target screening

The 211 therapeutic targets discovered for “QN” agarwood in its potential treatment of DKD were employed to construct a PPI network. The overlapping targets were subjected to analysis using the String platform and subsequently loaded into Cytoscape 3.7.1 software. The targets with a degree value exceeding 96 were identified as the core targets, with IL6, TNF, EGFR, and BCL-2 ranking among the top four targets based on their degree values (Fig. 3C). The above targets were postulated to potentially serve as the primary targets of “QN” agarwood in its therapeutic application for the treatment of DKD. The size and color of the icon change based on the degree value. As the degree value increases, the shape becomes larger, and the color becomes redder. The selection of the top 3 active ingredients was determined by the degree value in the “Active Ingredient-Target” network, resulting in the inclusion of “QN”7: 2-[2-(4-hydroxyphenyl)ethyl]chromone, “QN”2: 2-(2-phenylethyl)chromone, and “QN”9: 6-methoxy-2-[2-(4-methoxyphenyl)ethyl]chromone (Fig. 3B), and it is hypothesized that the above components may be the primary active ingredients of “QN” agarwood in addressing DKD.

### GO and KEGG pathway enrichment analysis

The intersection targets were uploaded to the David platform, where the Identifier was set as OFFICIAL-GENE-SYMBOL, and the species was selected as HOMO species. In this study, a total of 966 biological processes (BP) terms, 118 cellular composition (CC) terms, and 178 molecular function (MF) terms were identified. The top 10 terms from each category were chosen for visualization (Fig. 3D). By conducting KEGG pathway enrichment analysis, a total of 150 pathways were identified, and the top 20 terms were selected for visualization (Fig. 3E). The therapeutic targets of “QN” agarwood for the treatment of DKD predominantly focus on pathways associated with cancer, EGFR tyrosine kinase inhibitor resistance, and the HIF-1 signaling pathway. Notably, the pathway of EGFR signaling pathway demonstrates a significant relevance to DKD [23].

### Active ingredient binding to therapeutic targets was validated using molecular docking

**Fig. 4:**
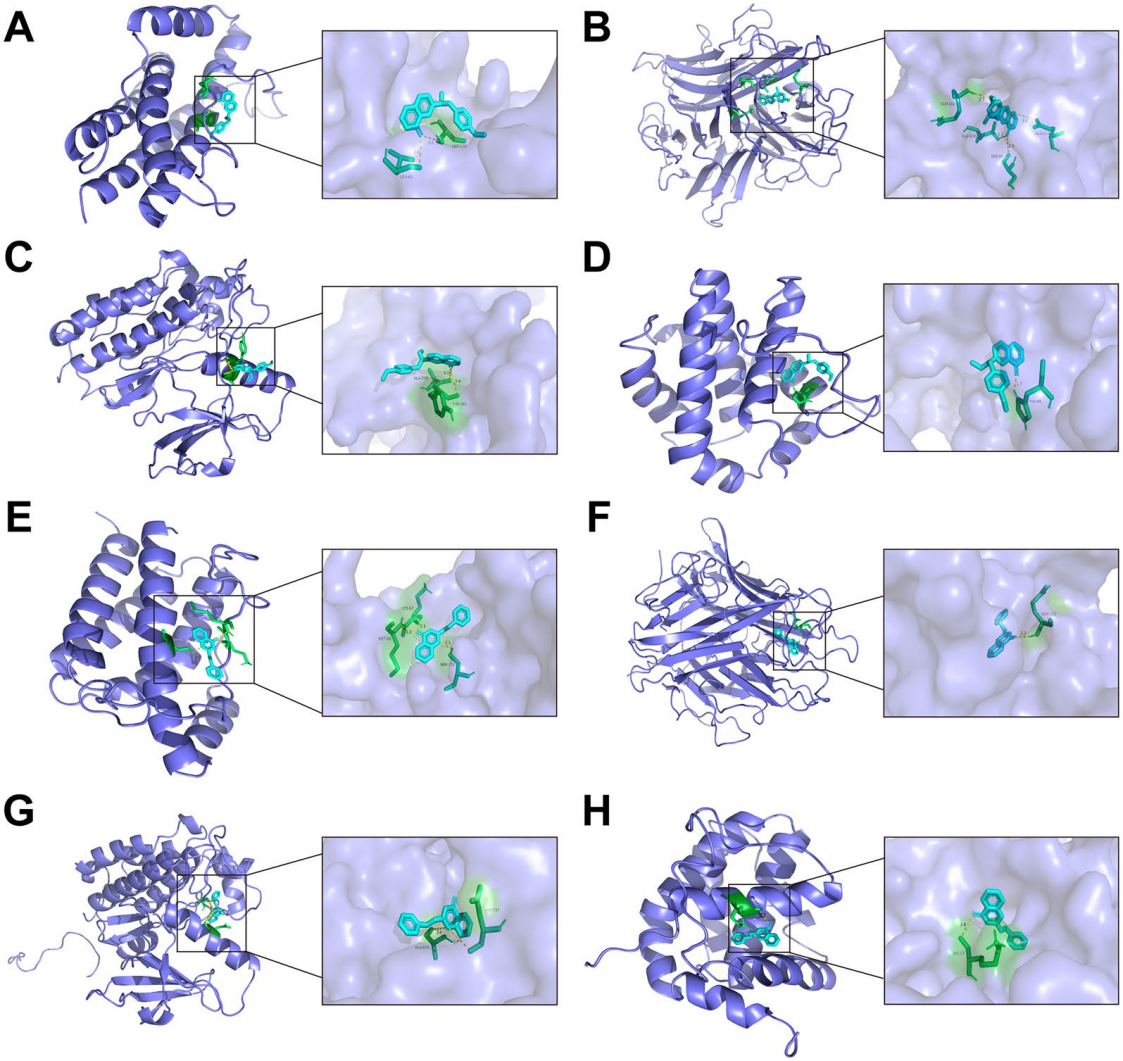
Docking analysis: assessing binding affinity of hub targets predicted by network pharmacology. Docking panorama and local magnification of 6-methoxy-2-[2-(4-methoxyphenyl)ethyl]chromone with IL6 (**A**), TNF (**B**), EGFR (**C**), BCL-2 (**D**); Docking panorama and local magnification of 2-(2-phenylethyl)chromone with Il6 (**E**), TNF (**F**), EGFR (**G**), BCL-2 (**H**)

Autodock is an advanced software designed for molecular modeling and simulation in the field of life sciences, with a specific focus on protein structure and function research and drug discovery applications [17]. Molecular docking was utilized to explore the binding affinities between the four core targets (IL6, TNF, EGFR, BCL-2) and the top three active ingredients (2-[2-(4-hydroxyphenyl) ethyl]chromone, 2-(2-phenylethyl)chromone, 6-methoxy-2-[2-(4-methoxyphenyl)ethyl]chromone). Due to the unavailability of a relevant crystal structure for 2-[2-(4-hydroxyphenyl)ethyl]chromone in the PDB database, only the remaining two compounds were subjected to docking analysis. The findings of the docking experiments are displayed in Table 3. The binding energies between the two active ingredients and the four key targets of “QN” agarwood were found to be below 5 kcal/mol. This suggests that the active components of “QN” agarwood exhibit strong binding ability with the core targets, indicating their potential effectiveness in exerting anti-DKD effects. A subset of the docking results was rendered using Pymol software, and the corresponding visualization is depicted in Fig. 4.

**Table 3:** Molecular docking

Active ingredients	Energy (kcal/mol)			
	IL6	TNF	EGFR	BCL2
2-(2-phenylethyl)chromone	− 8.47	− 6.15	− 5.98	− 9.15
6-methoxy-2-[2-(4-methoxyphenyl)ethyl]chromone)	− 7.01	− 5.79	− 5.07	− 7.9

### “QN” agarwood downregulation of EGFR improves podocyte autophagy

**Fig. 5:**
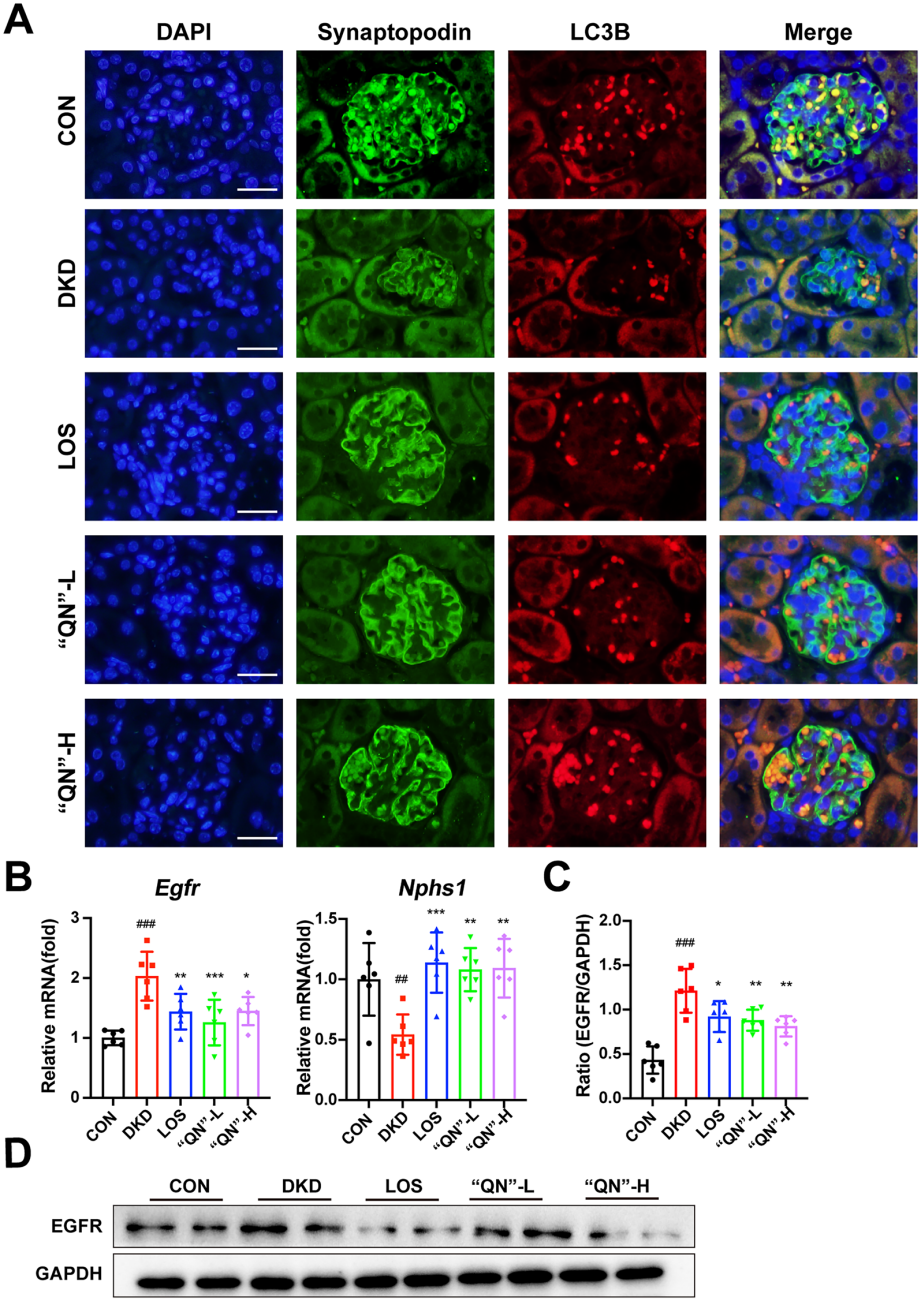
“QN” agarwood downregulated EGFR and improved autophagy in podocytes. **A** Immunofluorescence co-localization staining of the marker synaptopodin of podocytes with LC3B (a marker of autophagy), Scale bar = 100X. **B** The expression levels of *Egfr*, and *Nphs1* mRNA in the renal tissue. **C**,** D** WB of EGFR in the kidney. ^*##*^*p* < 0.01, ^*###*^*p* < 0.001 versus CON group; ^*^p < 0.05, ^**^p < 0.01, ^***^p < 0.001 versus DKD group

EGFR exhibits widespread expression throughout the mammalian kidney, including podocytes [24, 25]. Activation of EGFR activation plays a critical role in the pathways involved in podocyte damage and loss during the progression of DKD [26]. In laboratory models of DKD, renal EGFR signaling is activated, and inhibiting of EGFR signaling prevents the progression of DKD. Specifically, activation of EGFR signaling in podocytes specifically has been demonstrated to inhibit podocyte autophagy [27]. Immunofluorescence co-localization of Synaptopodin with LC3B revealed that podocyte morphology remained relatively intact, the number of podocytes was increased in diabetic mice after administration of “QN” agarwood. In addition, there was an upregulation in the expression of the autophagy marker LC3B (Fig. 5A). The q-PCR and WB analyses yielded additional evidence supporting the downregulation of EGFR expression and the concurrent increment in the number of podocytes by “QN” agarwood. In summary, the utilization of “QN” agarwood leads to the downregulation of EGFR expression and mitigates the inhibition of autophagy in podocytes, consequently providing protective effects for them. This beneficial effect contributes to the postponement of DKD progression.

## Discussion

While advancements in diabetes management have contributed significantly to enhanced outcomes for individuals with DKD, the pursuit of innovative therapeutic approaches for the prevention, treatment, and reversal of DKD remains an unmet need [28]. Excessive protein excretion and a decline in glomerular filtration rate are the primary clinical manifestations observed in DKD. The glomerular filtration membrane consists of endothelial cells, basement membrane, and podocytes arranged in the order from inner to outer layers. Among them, the outermost layer composed of podocytes and their specialized structures called slit diaphragms (SD) serves as the primary barrier in glomerular filtration, playing a crucial role in the formation of proteinuria. Accumulating evidence indicates that the impairment and loss of podocytes are critical contributors to the pathogenesis of DKD [29, 30]. The downregulation of podocyte markers, such as Synaptopodin, has been identified as an indicator of foot cell damage and dysfunction [31]. Autophagy involves three distinct stages, namely autophagosome formation, autophagosome-lysosome fusion, and content degradation, which are tightly regulated by a coordinated interplay of various autophagy-related genes, including LC3B [32]. Diabetic patients with significant albuminuria exhibit inadequate podocyte autophagy, and this compromised autophagic process further exacerbates proteinuria in DKD [33]. Podocyte injury, characterized by epithelial-mesenchymal transition, detachment, and apoptosis, is primarily attributed to the downregulation of podocyte autophagy [34]. Hence, interventions aimed at restoring autophagy levels in podocytes during pathological conditions hold significant importance in the management of kidney diseases [35–37].

There is a significant body of literature demonstrating an increased interest in herbal products, driven by the recognition that multi-ingredient formulations offer enhanced efficacy for managing complex diseases [38, 39]. Traditional Chinese Medicine has shown remarkable effectiveness in alleviating clinical symptoms, slowing the progression of DKD, enhancing renal function, and improving patients' quality of life. In this study, we conducted a comprehensive evaluation of the pharmacodynamic effects of “QN” agarwood. During diabetes mellitus progression, renal function significantly deteriorates in mice, evidenced by elevated albuminuria, Scr, and BUN levels, along with morphological changes in renal histoarchitecture. Our findings highlight the potential of “QN” agarwood treatment in attenuation of renal injury, inflammatory infiltration, and fibrosis.

The emergence and advancement of network pharmacology are intricately linked to the investigations conducted in TCM research [17]. The examination of the effective mechanisms behind the diverse, multi-faceted, and comprehensive therapeutic effects of traditional Chinese medicine is highly compatible with the utilization of network pharmacology. Network pharmacology is utilized to examine the interaction pathways between drugs and proteins or genes, offering a comprehensive network-based view of the intricate connections between biological systems, drugs, and diseases [40]. It is postulated that “QN” agarwood may regulate key factors such as IL6, TNF, EGFR, and BCL-2, contributing to its efficacy in DKD treatment. IL6, a versatile cytokine involved in immune and inflammatory responses, plays an important role in the development of DKD in individuals with diabetes [41]. Macrophage recruitment is intricately linked to the progression of DKD, TNF-α is produced by macrophages, and macrophage production of TNF-α plays an important role in diabetic kidney damage [42]. EGFR is involved in regulating both physiological and pathophysiological functions within the adult kidney. Prolonged activation of EGFR receptors plays a substantial pathological role in the advancement and worsening of DKD. Furthermore, compelling evidence suggests that EGFR activation leads to a decline in autophagy levels in podocytes [43].

In the present study, we have elucidated significant findings on “QN” agarwood-treated diabetic mice, underscoring the academic rigor and scientific relevance of our investigation. We observed downregulation of EGFR, upregulation of LC3B, and a substantial increase in the expression of synaptophysin-an actin-binding protein intricately positioned within the podocyte cytoskeleton in “QN” agarwood-treated diabetic mice. However, what kind of components play a role in “QN” agarwood needs to be verified by further experiments. It is essential to acknowledge potential omissions in the recruitment process of the TCM database and disease target-related information, as well as the possibility of errors introduced by big data enrichment analysis and computation. It is crucial to extend the application of “QN” agarwood to hypertensive nephropathy, lupus nephritis, and gouty nephropathy models in the future. Moreover, lacking predictive value for human nephrotoxicity due to species-specific differences [44]. Consequently, it is imperative to conduct further experimental validation to enhance our understanding of the biological characteristics and mechanisms.

## Conclusions

In summary, our study provides evidence on the beneficial effects of “QN” agarwood on podocyte damage in DKD. Furthermore, “QN” agarwood promotes podocyte autophagy at least in part by inhibiting the EGFR signaling pathway. Consequently, “QN” agarwood might be a potential candidate agent for DKD treatment.

## Data Availability

The data used to support the results of this study are available from the corresponding author upon reasonable request.

## Abbreviations

“QN” Agarwood: “Qi-Nan” Agarwood
DKD: Diabetic kidney disease
HFD: High-fat diet
STZ: Streptozotocin
CMC-Na: Carboxymethylcellulose sodium
TGF-β1: Transforming growth factor β1
Ccl2: C-C motif chemokine ligand 2
FN: Fibronectin
H&E: Hematoxylin–eosin staining
IHC: Immunohistochemistry
IF: Immunofluorescence
IL6: Interleukin 6
KEGG: Kyoto encyclopedia of genes and genomes
GO: Gene ontology
Scr: Serum creatinine
BUN: Blood urea nitrogen
OMIM: Online Mendelian inheritance in man
PPI: Protein–protein interaction
EGFR: Epidermal growth factor receptor
